# Association of Early Life Exposure to Phthalates With Obesity and Cardiometabolic Traits in Childhood: Sex Specific Associations

**DOI:** 10.3389/fpubh.2018.00327

**Published:** 2018-11-27

**Authors:** Marina Vafeiadi, Antonis Myridakis, Theano Roumeliotaki, Katerina Margetaki, Georgia Chalkiadaki, Eirini Dermitzaki, Maria Venihaki, Katerina Sarri, Maria Vassilaki, Vasiliki Leventakou, Euripides G. Stephanou, Manolis Kogevinas, Leda Chatzi

**Affiliations:** ^1^Department of Social Medicine, Faculty of Medicine, University of Crete, Heraklion, Greece; ^2^Environmental Chemical Processes Laboratory (ECPL), Department of Chemistry, University of Crete, Heraklion, Greece; ^3^Department of Clinical Chemistry, School of Medicine, University of Crete, Heraklion, Greece; ^4^Department of Health Sciences Research, Mayo Clinic, Rochester, MN, United States; ^5^Barcelona Institute for Global Health (ISGlobal), Barcelona, Spain; ^6^Hospital del Mar Research Institute (IMIM), Barcelona, Spain; ^7^Department of Preventive Medicine, Keck School of Medicine, University of Southern California, Los Angeles, MN, United States; ^8^Department of Genetics & Cell Biology, Faculty of Health, Medicine and Life Sciences, Maastricht University, Maastricht, Netherlands

**Keywords:** phthalates, pregnancy, children, obesity, cardiometabolic risk

## Abstract

Few studies have investigated longitudinal associations between early life phthalate exposure and subsequent obesity and cardiovascular risks in children with inconsistent results. We aimed to evaluate the associations between phthalate exposure during gestation and childhood with offspring obesity and cardiometabolic risk factors in 500 mother-child pairs from the Rhea pregnancy cohort in Crete, Greece. Seven phthalate metabolites [monoethyl phthalate (MEP), mono-n-butyl phthalate (MnBP), mono-isobutyl phthalate (MiBP), monobenzyl phthalate (MBzP), mono(2-ethylhexyl) phthalate (MEHP), mono(2-ethyl-5-hydroxyhexyl) phthalate (MEHHP), and mono(2-ethyl-5-oxohexyl) phthalate (MEOHP)] were quantified in spot urine samples collected from mothers (1st trimester) and their children at 4 years of age. We calculated the molar sum of DEHP metabolites (MEHP, MEHHP, MEOHP). We measured child weight, height, waist circumference, skinfold thicknesses, blood pressure (BP), and lipids at 4 and 6 years and leptin, adiponectin, and C-reactive protein at 4 years. We used generalized estimating equations to examine associations at each age and tested for interaction by sex. Child exposure to phthalate metabolites was associated with lower BMI z-scores in boys and higher BMI z-scores in girls. Each 10-fold increase in ΣDEHP was associated with a change in waist circumference of −2.6 cm (95% CI: −4.72, −0.48) in boys vs. 2.14 cm (95% CI: −0.14, 4.43) in girls (*p*-sex interaction = 0.003) and a change in waist-to-height ratio of −0.01 (95% CI: −0.03, 0.01) in boys vs. 0.02 (95% CI: 0.01, 0.04) in girls (*p*-sex interaction = 0.006). Phthalate metabolite concentrations at age 4 were negatively associated with systolic and diastolic blood pressure. MEP was associated with lower systolic BP z-scores (adj. β = −0.22; 95% CI: −0.36, −0.08) at 4 years. MnBP and MBzP were associated with lower diastolic BP z-scores (adj. β = −0.13; 95%CI: −0.23, −0.04, and adj. β = −0.11; 95% CI: −0.21, −0.01, respectively). A 10-fold increase in MiBP was associated with 4.4% higher total cholesterol levels (95% CI: 0.2, 8.7). Prenatal phthalate exposure was not consistently associated with child adiposity and cardiometabolic measures. Our findings suggest that early life phthalate exposure may affect child growth and adiposity in a sex-specific manner and depends on the timing of exposure.

## Introduction

Childhood obesity is a risk factor for obesity-related health outcomes such as cardiovascular and other chronic diseases later in life and one of the most common public health problems and challenges globally. The global prevalence of childhood overweight and obesity has increased over the last three decades to 124 million in 2016 (1) and if current trends continue, the World Health Organization (WHO) predicts that 70 million young children will be overweight or obese by 2025 (2). In Greece, the leading country in the childhood obesity epidemic worldwide, recent figures show that more than 40% of children at the age of 5–17 years are overweight or obese (3). Obesity and associated risk factors, such as high blood pressure and dyslipidemia, in childhood can induce changes in metabolism and contribute to the development of atherosclerosis in adulthood (4). Diet and physical inactivity are recognized causes, but exposure to environmental chemicals may also contribute to the pathogenesis of obesity and related cardiometabolic disorders (5, 6). *Obesogens*-chemicals that inappropriately regulate and promote lipid accumulation and adipogenesis-may contribute to obesity development, especially when exposure occurs during pregnancy and early life (7–9).

Phthalates, or diesters of phthalic acid, are a class of man-made chemicals used in a variety of common consumer and industrial products. Low-molecular weight phthalates (LMWP) are used as solvents and are typically found in medications and personal care items such as shampoos, deodorants, and lotions, while high-molecular weight phthalates (HMWP) are used in the manufacturing of flexible plastics for purposes such as vinyl flooring, adhesives, medical devices, and food packaging (10–12). Exposure to phthalates is almost ubiquitous (13) and may occur through dermal absorption, inhalation, or ingestion (14). Moreover, detectable levels of phthalate metabolites have been found in amniotic fluids and cord blood, indicating fetal exposure (12). In the Rhea mother-child cohort in Greece, Myridakis et al. found that daily intake of phthalate esters, calculated for 4 year old children, was lower than the corresponding daily intake for 2.5 year old children and that phthalate esters generally were assigned to combined exposure from plastic and diethyl phthalate from personal hygiene products/cosmetics (15, 16).

Phthalates are endocrine-disrupting chemicals with anti-androgenic and weakly estrogenic properties (17) and evidence from experimental studies suggests that phthalates may influence obesity through several mechanisms, including antithyroid hormone activities, and/or activation of peroxisome proliferator-activated receptors (PPARs), and epigenetic modulation (14, 18).

Results from cross-sectional studies in adults and children show that higher urinary phthalates concentrations are positively associated with adiposity and cardiometabolic markers (19–23). However, only a few longitudinal studies have examined the associations of early-life exposure to phthalate metabolites with childhood growth and obesity (24–32) and these studies have shown inconsistent results and sex-specific associations. *In utero* exposures were found to be associated with decreased BMI z-scores only in girls aged 4–7 years (25), or only in boys aged 4–7 years old (28, 29). A study in France that examined only boys found that prenatal phthalate exposure was associated with increased BMI at age 5 (26). Additionally, a US study that examined only girls found that exposure to phthalates at ages 6–8 were associated with a predicted decrease in BMI from the ages of 7–13 (31). Few studies have examined the effects of early life phthalate exposure on offspring cardiovascular traits other than adiposity. Valvi et al. (28) reported that prenatal exposure to phthalates was associated with lower systolic blood pressure z-scores at 4–7 years of age in girls but not in boys and Perng et al. (33) reported that concurrent exposure to phthalates was associated with lower cholesterol levels at 8–14 years of age.

In the present study, using data from the Rhea birth cohort, we assessed whether urinary phthalate metabolite concentrations in pregnant women and their children in early childhood were associated with measures of adiposity and a full range of cardiometabolic traits at ages 4–6 years and evaluated differences by child's sex.

## Materials and methods

### Subjects and study design

The present study is part of the Rhea Study, a prospective mother-child cohort examining a population sample of pregnant women and their children at the prefecture of Heraklion, Crete, Greece. Methods are described in detail elsewhere (34). Briefly, women (Greek and immigrants) who became pregnant during a period of 1 year starting in February 2007 were asked to participate in the study. The first contact was made at the time of the first comprehensive ultrasound examination (mean ± SD 11.96 ± 1.49 weeks) and several contacts followed (6th month of pregnancy, at birth, 9 months, 1st year, 4 and 6 years after birth). To be eligible for inclusion in the study, women had to have a good understanding of the Greek language and be older than 16 years of age. The study was approved by the ethics committee of the University Hospital in Heraklion, Crete, Greece, and all participants provided written informed consent after complete description of the study.

Of 1363 singleton live births in the Rhea study, phthalate concentrations were measured in spot urine samples collected in the first trimester of pregnancy from 260 mothers and 500 children at 4 years of age; 500 children had at least one measure of BMI between 4 and 6 years of age.

### Maternal and child phthalate biomarkers measurements

All spot urine samples were collected in sterile, polypropylene urine cups, aliquoted in 4 ml cryotube vials (Thermo Fisher Scientific, USA) and stored at −80°C. Analyses were performed at the Environmental Chemical Processes Laboratory (ECPL) in the Department of Chemistry of the University of Crete. An aliquot of each urine sample (1 mL) was analyzed for the determination of the following 7 phthalate metabolites, using a previously described analytical protocol (35): monoethyl phthalate (MEP), mono-n-butyl phthalate (MnBP), mono-isobutyl phthalate (MiBP), monobenzyl phthalate (MBzP), mono(2-ethylhexyl) phthalate (MEHP), mono(2-ethyl-5-hydroxyhexyl) phthalate (MEHHP), and mono(2-ethyl-5-oxohexyl) phthalate (MEOHP). Samples exceeding the upper limit of linearity were reanalyzed, diluted with nanopure water. Two quality control samples (spiked pooled urine) and two blank samples (synthetic urine) were analyzed with every forty six (46) urine samples. The amount of each sample was quantified by the standard curve performed in each assay. All samples were measured in duplicates. We calculated the molar sums of ΣDEHP metabolites (MEHP, MEHHP, and MEOHP) by dividing metabolite concentrations by their molecular weight (MW) and summing across. Method limit of detection (LOD) ranged from 0.01 to 2.5 ng/mL and samples below LOD were assigned the value of LOD/√2. To account for urine dilution, a second aliquot of 0.5 mL urine was analyzed for creatinine concentration using the OLYMPUS 2700 immunoassay system (Beckman Coulter, USA), and phthalate metabolites concentrations were divided by urinary creatinine levels (i.e., creatinine-adjusted concentrations, hereafter). All creatinine-adjusted concentrations were log_10_ transformed to obtain normal distributions, as the original distributions were right skewed.

### Child anthropometry

At 4 and 6 years, child anthropometry measures, including weight and length/height, waist circumference and skinfold thickness at four sites of the body (subscapular, triceps, suprailiac, and quadriceps) were obtained by specially trained research assistants following standard operating procedures. We calculated BMI (weight/height^2^) and converted raw values into sex- and age-specific standard deviation (SD) scores (z scores) by using internally generated growth reference curves (36). We analyzed child BMI z-score as a continuous outcome and in categories of overweight/obesity at 4 and 6 years according to the BMI cutoff points for sex and age proposed by the International Obesity Task Force (IOTF) definitions (37). The sum of the four aforementioned skinfolds was calculated as an indicator of subcutaneous fat. We also divided child waist circumference by height to calculate the waist-to-height ratio.

### Child cardiometabolic risk factors

At the 4 and 6 year examination, after 5 min rest in the seated position, trained research assistants measured systolic (SBP), and diastolic (DBP) blood pressure levels on the child's right arm using an automatic oscillometric device (Dinamap, Pro Care 400) with a cuff of appropriate size for arm circumference. Five measurements were made with 1 min intervals and the average of these measurements was used for analysis (38). We calculated age, sex, and height specific blood pressure SD scores. Non-fasting blood samples were collected from the children at the end of the visit in 10 mL BD gel separator vacutainers with the use of standard procedures, with samples immediately spun, separated, and frozen at −80°C. Analysis of lipids (total cholesterol and high-density lipoprotein cholesterol [HDL-C]) was performed by standard enzymatic methods (Medicon, Greece). Leptin, adiponectin, and CRP levels were measured at 4 years of age. All inter- and intra-assay coefficients of variation were <5.5%.

### Statistical analysis

Descriptive analyses of the study population characteristics, exposures, and outcomes were conducted. Generalized additive models (GAMs) were applied to explore the shape of the relationships between prenatal or early-childhood phthalate metabolite concentrations and outcomes under study. Linearity was assumed if the p-gain defined as the difference in normalized deviance between the GAM model and the linear model for the same exposure and outcome was >0.10. We used generalized estimating equations (GEEs) with a Gaussian or Poisson family specification and with exchangeable correlation structure to examine the associations between phthalate metabolite concentrations and repeated continuous and binary outcomes, respectively. We included interaction terms between the exposure variable and child age at examination to explore the possibility of age-specific associations. We used linear regression analyses to test whether phthalate metabolite concentrations were associated with serum leptin, adiponectin and CRP levels at 4 years. Due to non-normal distributions, we natural log-(ln)-transformed serum leptin, adiponectin, and CRP levels at 4 years, and total and HDL cholesterol at 4 and 6 years and present results as a % difference in each of these outcomes per 10-fold increase in exposure.

For every outcome variable, we first studied the association of interest in the crude model. To control for confounding, we considered maternal and child covariates that were of *a priori* interest as independent predictors of child adiposity and growth or that may be related to phthalate levels: maternal pre-pregnancy BMI (kg/m^2^), maternal age at birth (years), parity (primiparous, multiparous), maternal educational level [low level: ≤ 9 years of mandatory schooling, medium level: >9 years of schooling up to attending post-secondary school education and high level: attending university or having a university/technical college degree], smoking during pregnancy (never, ever), gestational weight gain [GWG (kg)], ethnic origin (Greek, non-Greek), residence (urban, rural), delivery type (vaginal delivery, cesarean section), delivery hospital (private, public), marital status (married, not married), and working during pregnancy (yes, no), breastfeeding (yes, no), gestational length, sex, age at outcome assessment, time watching television at 4 and 6 years (< 30 min, 1–2 h, ≥3 h per day), and child's BMI at 4 and 6 years (for models that used cardiometabolic risk factors as outcomes). We included covariates in the final models if they were associated with both exposure and any of the outcomes at *p*-value < 0.2 or if they modified the coefficient for the phthalate exposure variable by >10%. To assess the potential modifying effects of child sex (male, female), we included interaction terms between the exposure variable and sex in the models and stratified the sample, on the basis of prior studies (25, 28, 29). We also performed sensitivity analysis in order to explore remaining confounding. In particular, we repeated the analyses excluding children who had been born preterm (< 37 gestational weeks) or at low birth weight (< 2,500 g). Statistical significance was defined by an alpha level of 0.10 for interaction terms and of 0.05 for all other effect estimates. Analyses were conducted using STATA software, version 13.0 (Statacorp, College Station, TX).

## Results

Maternal and child characteristics of the study population are presented in Table 1. Participating mothers were predominantly of Greek origin, had a mean (±SD) age of 29.5 (± 5.1) years at delivery, about half of them had medium educational level (52%) and were multiparous (56%). Before pregnancy, 20% of mothers were overweight, the majority of mothers (86%) initiated breastfeeding and the mean (±SD) length of breastfeeding was 4.1 (± 4.3) months. Fifty-six percent of the children included in the analysis were boys, their mean (±SD) birth weight was 3,203 (± 462.8) g and the average (±SD) gestation was 38.2 (± 1.5) weeks. Twenty four percent of the children were overweight at 4 years and 22% at 6 years.

**Table 1:** Maternal and child characteristics, Mother-child cohort “Rhea” in Crete, Greece (*n* = 500).

	***N***	**% or Mean ±SD**
**MATERNAL CHARACTERISTICS**		
Maternal age (years)	497	29.5 ± 5.1
Pre-pregnancy BMI (kg/m^2^)		
Underweight (< 18.5)	23	4.9
Normal (≥18.5–25)	291	61.8
Overweight (≥25–30)	96	20.4
Obese (≥30)	61	13.0
Gestational weight gain (kg)	406	13.5 ± 5.7
Ethnic origin (Greek, %)	469	94.8
Education		
Low	89	18.2
Medium	255	52.4
High	143	29.4
Parity (multiparous, %)	269	56.3
Smoking during pregnancy (yes, %)	166	36.1
Breastfeeding (yes, %)	406	86.2
Breastfeeding (months)	471	4.1 ± 4.3
**CHILD CHARACTERISTICS**		
**INFANCY**		
Sex (boy, %)	279	55.5
Birth weight (g)	479	3203 ± 462.8
Gestational age (completed weeks)	475	38.2 ± 1.5
**AT 4 YEARS OF AGE**		
Weight (kg)	500	18.4 ± 3.1
Height (cm)	500	105.3 ± 4.3
BMI (kg/m^2^)	500	16.5 ± 1.9
Overweight/obese (yes, %)	120	23.9
Waist circumference (cm)	500	53.8 ± 5.0
Sum of skinfolds (mm)	463	41.0 ± 13.9
Systolic blood pressure (mmHg)	388	90.5 ± 7.5
Diastolic blood pressure (mmHg)	388	53.4 ± 5.3
Total cholesterol (mg/dL)	475	155.6 ± 27.7
HDL-C (mg/dL)	475	49.5 ± 11.0
C-reactive protein (mg/dl)	438	0.2 ± 0.7
Leptin (ng/ml)	479	3.1 ± 3.9
Adiponectin (ug/ml)	461	15.5 ± 8.8
**AT 6 YEARS OF AGE**		
Weight (kg)	331	25.1 ± 5.4
Height (m)	331	1.2 ± 0.1
BMI (kg/m^2^)	331	17.2 ± 2.8
Overweight/obese (yes, %)	74	22.4
Waist circumference (cm)	330	59.2 ± 4.5
Sum of skinfolds (mm)	281	47.9 ± 47.3
Systolic blood pressure (mmHg)	329	105.1 ± 89.9
Diastolic blood pressure (mmHg)	329	65.1 ± 93.6
Total cholesterol (mg/dL)	312	161.7 ± 23.9
HDL-C (mg/dL)	312	58.2 ± 11.9

The distribution of urinary phthalate metabolite concentrations in the pregnant women and their children is shown in Table 2. The majority of samples show detectable concentrations of creatinine-corrected metabolites, with the lowest detection in MEOHP in the mothers. The phthalate metabolite with the highest concentrations in maternal and child urine was MEP. Spearman correlations of creatinine-corrected individual phthalate metabolites were weakly to highly correlated (range 0.11–0.95) within each time period while concentrations between prenatal and child measures were not consistently correlated with each other (data not shown).

**Table 2:** Maternal and children urinary concentrations of phthalate metabolites (μg/g creatinine), Mother-child cohort “Rhea” in Crete, Greece.

					**Percentile**		
	**LOD**	**%>LOD**	**GM (GSD)**	**min**	**25th**	**50th**	**75th**	**max**
**Prenatal (*****n*** = **230)**								
MEP	1.26	100	141.1 (3.7)	4.8	55.0	130.6	389.5	3993.8
MnBP	2.48	98.1	37.1 (2.4)	0.7	23.3	38.6	59.6	720.0
MiBP	2.09	95.8	33.5 (3.1)	2.5	16.8	33.5	61.3	48799.3
MBzP	1.76	96.6	23.1 (2.9)	0.4	13.2	25.6	39.5	5095.4
MEHP	2.20	92.4	16.0 (3.1)	0.4	8.9	16.5	30.0	2935.4
MEHHP	0.82	91.7	7.1 (2.5)	0.9	3.9	7.0	12.1	132.0
MEOHP	0.94	72.0	6.9 (3.3)	0.7	2.8	7.3	13.7	2765.3
ΣDEHP^a^	–	–	0.1 (2.6)	0.02	0.1	0.1	0.2	20.2
**Child (*****n*** = **500)**								
MEP	0.40	99.8	62.7 (2.9)	0.9	31.2	53.7	111.5	17611.8
MnBP	0.25	93.4	21.7 (4.6)	0.2	15.5	28.1	53.0	695.0
MiBP	0.41	96.8	41.1 (3.3)	0.2	28.9	49.3	79.4	671.4
MBzP	0.02	99.0	7.4 (3.2)	0.0	3.8	7.0	13.6	313.6
MEHP	0.84	100	11.1 (2.3)	1.7	6.1	10.5	19.4	300.7
MEHHP	0.01	100	43.3 (2.3)	0.2	26.9	40.9	68.1	2246.1
MEOHP	0.18	100	34.6 (2.4)	0.4	21.3	35.4	58.3	1652.7
ΣDEHP^a^	–	–	0.3 (2.1)	0.01	0.2	0.3	0.5	14.1

*ΣDEHP: molar sum of MEHP, MEHHP, MEOHP*.

a *∑DEHP is expressed as micromoles/g*.

GAMs examining the shape of the relationships of phthalate metabolite concentrations at age 4 with adiposity indicators and cardiometabolic risk factors at all ages showed no significant departures from linearity (see Figure S1 for blood pressure at age 4).

Prenatal MnBP was associated with a change in waist-to-height ratio at ages 4 to 6 years of 0.19 (95% CI: 0.11, 0.27) in boys vs. −0.01 (95% CI: −0.03, 0.01) in girls (p-sex interaction < 0.001; see Table S1). MiBP was associated with lower diastolic BP z-score overall (adj. β = −0.2; 95% CI: −0.37, −0.03) and in boys (adj. β = −0.26; 95% CI: −0.48, −0.04; Table 3). There was a marginally significant association between ΣDEHP and systolic (adj. β = −0.16; 95% CI: −0.35, 0.03) and diastolic (adj. β = −0.12; 95% CI: −0.25, 0.01) BP z-score. There was a significant interaction of prenatal MEP and age (*P*-interaction < 0.05); the association between MEP and HDL-C was negative at 4 years (adj. β = −5.8; 95% CI: −11.3, 0.0) and positive but not statistically significant at 6 years of age (adj. β = 2.8; 95% CI: −3.6, 9.7).

**Table 3:** Sex-stratified associations between prenatal urinary individual and summed ΣDEHP phthalate metabolites (log_10_ Transformed, in μg/g Creatinine) with lipids and blood pressure levels in children aged 4–6 years.

		**All**		**Boys**		**Girls**
	***n***	β **(95% CI)**	***n***	β **(95% CI)**	***n***	β **(95% CI)**	***p*****-sex interaction**
Systolic BP z-score							
MEP	206	0.02 (−0.13, 0.18)	117	−0.04 (−0.24, 0.15)	89	0.13 (−0.12, 0.38)	0.176
MnBP	195	−0.08 (−0.29, 0.13)	114	0.01 (−0.25, 0.26)	81	−0.18 (−0.54, 0.18)	0.429
MiBP	202	−0.03 (−0.28, 0.22)	116	−0.16 (−0.49, 0.17)	86	0.17 (−0.2, 0.54)	0.136
MBzP	174	0.06 (−0.19, 0.32)	98	0.18 (−0.12, 0.47)	76	−0.23 (−0.7, 0.25)	0.213
ΣDEHP	189	−0.16 (−0.35, 0.03)	111	−0.09 (−0.31, 0.13)	78	−0.28 (−0.65, 0.1)	0.489
Diastolic BP z-score							
MEP	206	−0.02 (−0.13, 0.09)	117	−0.07 (−0.2, 0.07)	89	0.04 (−0.13, 0.21)	0.201
MnBP	195	−0.08 (−0.22, 0.07)	114	−0.04 (−0.21, 0.14)	81	−0.09 (−0.34, 0.15)	0.890
MiBP	202	−**0.2 (**−**0.37**, −**0.03)**	116	−**0.26 (**−**0.48**, −**0.04)**	86	−0.08 (−0.33, 0.17)	0.266
MBzP	174	0.04 (−0.14, 0.22)	98	0.1 (−0.1, 0.31)	76	−0.06 (−0.39, 0.27)	0.372
ΣDEHP	189	−0.12 (−0.25, 0.01)	111	−0.09 (−0.24, 0.05)	78	−0.17 (−0.42, 0.08)	0.636
Total Cholesterol		% Change (95% CI)		% Change (95% CI)		% Change (95% CI)	*p*-sex interaction
MEP	208	−1.0 (−4.7, 2.8)	114	−2.5 (−7.3, 2.6)	94	0.9 (−4.5, 6.7)	0.420
MnBP	197	1.9 (−2.3, 6.4)	111	−1.5 (−7.5, 4.8)	86	4.4 (−1.5, 10.6)	0.155
MiBP	202	1.4 (−4.3, 7.5)	113	−2.0 (−9.8, 6.4)	89	5.1 (−2.9, 13.7)	0.277
MBzP	178	1.8 (−4.4, 8.3)	97	−1.4 (−9, 6.9)	81	7.9 (−2, 18.8)	0.129
ΣDEHP	192	2.8 (−1.8, 7.7)	108	−0.1 (−5.5, 5.6)	84	9.1 (0.7, 18.2)	0.074
HDL-C
MEP	208	−2.0 (−7.1, 3.3)^*^	114	−3.9 (−9.8, 2.4)	94	−0.8 (−8.9, 7.9)	0.456
MnBP	197	−3.2 (−9.0, 3.0)	111	−2.5 (−10.1, 5.7)	86	−4.9 (−13.1, 4.1)	0.797
MiBP	202	1.6 (−6.4, 10.2)	113	−2.3 (−12, 8.4)	89	7.2 (−5.1, 21.1)	0.427
MBzP	178	4.7 (−4.0, 14.1)	97	4.2 (−5.5, 14.8)	81	6.7 (−8.1, 23.8)	0.571
ΣDEHP	192	1.2 (−5.1, 7.8)	108	0.6 (−6.0, 7.6)	84	0.3 (−11.5, 13.8)	0.960

*ΣDEHP: molar sum of MEHP, MEHHP, MEOHP. Total and HDL Cholesterol were log transformed to normalize their distributions. We calculated percent change by exponentiating beta coefficients, subtracting by 1 and multiplying by 100. All models are adjusted for child sex, exact age at examination, and maternal characteristics (age at delivery, parity, education, pre-pregnancy BMI, and smoking in pregnancy). Statistically significant associations (P < 0.05) are displayed in bold type*.

* *Interaction of exposure variable with child age at examination is statistically significant (P < 0.10)*.

Child sex modified the relationships between MnBP and ΣDEHP and CRP at 4 years (**Table 5**). A 10-fold increase in prenatal MnBP was associated with −49.1% (95% CI: −76.7, 11.2) lower and 144.3% (95% CI: 26.0, 373.8; *p*-sex interaction = 0.001) higher CRP levels in boys and girls, respectively (*p*-sex interaction = 0.001; **Table 5**). Associations of prenatal ΣDEHP with CRP were negative in boys (adj. β = −22.8; 95% CI: −58.8, 44.7) and positive in girls (adj. β = 116; 95% CI: −18.9, 475.1; *p*-sex interaction = 0.053) but not statistically significant. There were no other associations with adiposity indicators and cardiometabolic risk factors detected from the prenatal exposure period and we did not observe any modification of associations by sex.

**Figure 1:**
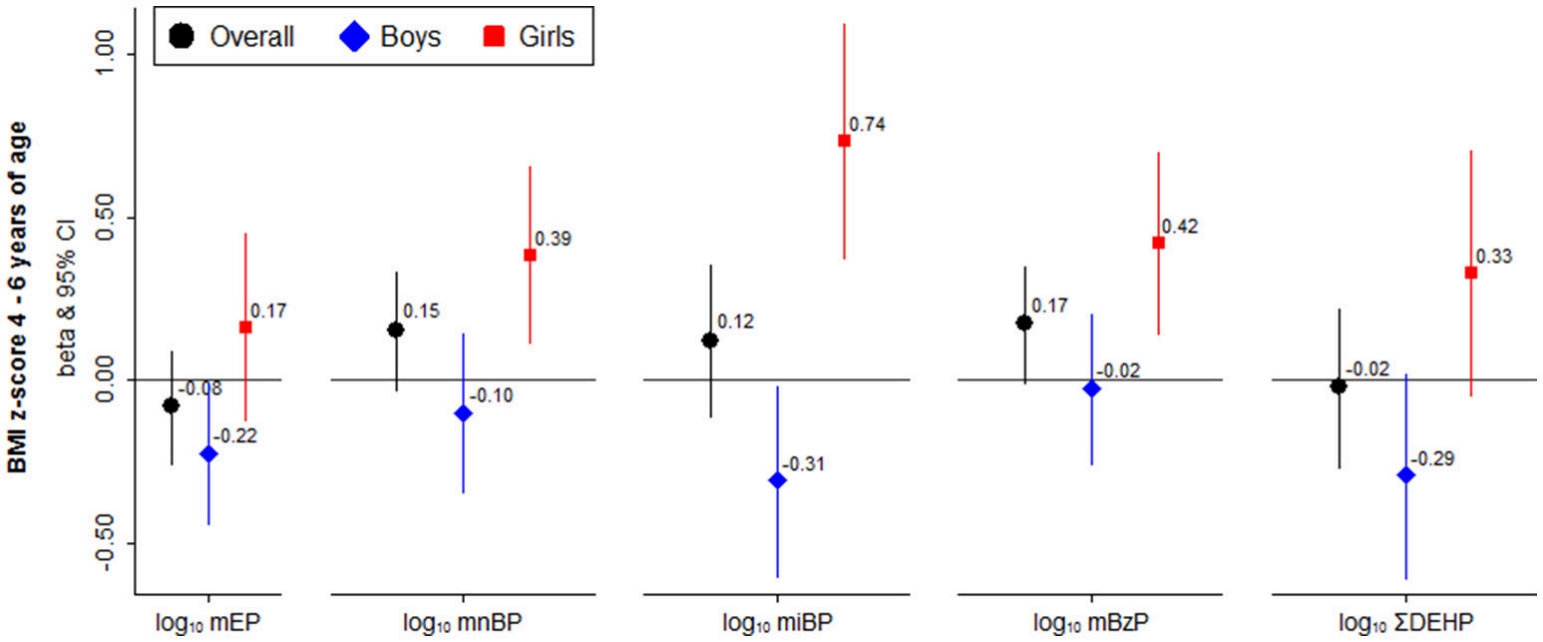
Sex-stratified associations between child urinary individual and summed ΣDEHP phthalate metabolites (log_10_ Transformed, in μg/g Creatinine) with child BMI z scores at 4 to 6 years of age. All models are adjusted for child sex, exact age at examination, and maternal characteristics (age at delivery, parity, education, pre-pregnancy BMI, and smoking in pregnancy).

Overall, creatinine-adjusted phthalate metabolite concentrations at age 4 were not significantly associated with BMI z-scores at 4 to 6 years of age (Figure 1 and Table S2). However, we detected sex-specific associations with respect to postnatal exposure. Child urinary phthalate metabolite concentrations were negatively associated with BMI z-scores in boys and positively in girls at ages 4 to 6 (Figure 1). MEP and MnBP were associated with lower BMI z-scores in boys (adj. β = −0.22; 95% CI: −0.44, −0.01 and adj. β = −0.1; 95% CI: −0.35, −0.15, respectively) and with higher BMI z-scores in girls (adj. β = 0.17; 95% CI: −0.12, 0.45 and adj. β = 0.39; 95% CI: 0.11, 0.66, respectively; *p*-sex interaction = 0.051 and 0.010, respectively; Table S2). Each 10-fold increase in MiBP was associated with a change in BMI z-score of −0.31 (95% CI: −0.6, −0.02) in boys vs. 0.74 (95% CI: 0.37, 1.1) in girls (*p*-sex interaction < 0.001). Similar associations were observed for ΣDEHP (Figure 1 and Table S2). When we analyzed dichotomous outcomes, results for overweight were consistent with those for BMI z-scores (data not shown).

Similarly, child urinary phthalate metabolite concentrations were negatively associated with waist circumference, sum of skinfolds and waist-to-height ratio in boys and positively in girls at age 4 and 6 (Table S2). For example, each 10-fold increase of ΣDEHP was associated with a change in waist circumference of −2.6 (95% CI: −4.72, −0.48) in boys vs. 2.14 (95% CI: −0.14, 4.43) in girls (*p*-sex interaction = 0.003) and a change in waist-to-height ratio of −0.01 (95% CI: −0.03, 0.01) in boys vs. 0.02 (95% CI: 0.01, 0.04) in girls (*p*-sex interaction = 0.006).

We observed consistent negative associations between phthalate metabolite concentrations at age 4 and systolic and diastolic blood pressure at all ages, overall and in girls and boys separately (Table 4). For example, MEP was associated with lower systolic BP z-score (adj. β = −0.22; 95% CI: −0.36, −0.08). MnBP and MBzP were associated with lower diastolic BP z-score (adj. β = −0.13; 95%CI: −0.23, −0.04 and adj. β = −0.11; 95% CI: −0.21, −0.01, respectively). A 10-fold increase in MiBP was associated with 4.4% higher total cholesterol levels (95% CI: 0.2, 8.7).

**Table 4:** Sex-stratified associations between child urinary individual and summed ΣDEHP phthalate metabolites (log_10_ Transformed, in μg/g Creatinine) with lipids and blood pressure levels in children aged 4–6 years.

		**All**		**Boys**		**Girls**		
	***n***	**β (95% CI)**	***n***	**β (95% CI)**	***n***	**β (95% CI)**	***p*-sex interaction**
Systolic BP Z-score							
MEP	409	−**0.22 (**−**0.36**, −**0.08)**	229	−**0.23 (**−**0.4**, −**0.05)**	180	−0.18 (−0.41, 0.05)	0.829
MnBP	385	−0.09 (−0.24, 0.06)	213	−0.09 (−0.3, 0.11)	172	−0.11 (−0.33, 0.11)	0.726
MiBP	399	0.05 (−0.14, 0.24)	223	0.01 (−0.23, 0.24)	176	0.03 (−0.27, 0.34)	0.872
MBzP	404	−0.01 (−0.16, 0.14)	228	−0.03 (−0.22, 0.16)	176	0.02 (−0.22, 0.26)	0.846
ΣDEHP	409	−0.1 (−0.3, 0.1)	229	−0.15 (−0.42, 0.11)	180	−0.06 (−0.37, 0.24)	0.848
Diastolic BP Z-score							
MEP	409	−0.09 (−0.18, 0.01)	229	−0.06 (−0.18, 0.05)	180	−0.12 (−0.27, 0.04)	0.518
MnBP	385	−**0.13 (**−**0.23**, −**0.04)**	213	−**0.16 (**−**0.29**, −**0.02)**	172	−0.13 (−0.27, 0.02)	0.977
MiBP	399	−0.04 (−0.16, 0.09)	223	−0.1 (−0.25, 0.05)	176	−0.01 (−0.21, 0.2)	0.515
MBzP	404	−**0.11 (**−**0.21**, −**0.01)**	228	−0.1 (−0.22, 0.03)	176	−0.14 (−0.3, 0.02)	0.538
ΣDEHP	409	−0.04 (−0.18, 0.09)	229	−0.14 (−0.31, 0.04)	180	0.02 (−0.19, 0.23)	0.275
Total Cholesterol		% Change (95% CI)		% Change (95% CI)		% Change (95% CI)	*p*-sex interaction
MEP	436	−0.1 (−3.1, 3)	243	1.5 (−2.6, 5.7)	193	−3.6 (−8.1, 1.2)	0.128
MnBP	408	0.9 (−2.3, 4.2)	225	1.5 (−3.1, 6.2)	183	0.5 (−3.8, 5.1)	0.966
MiBP	423	**4.4 (0.2, 8.7)**	235	2.3 (−3.1, 8)	188	**7.6 (1.1, 14.6)**	0.248
MBzP	431	2.7 (−0.6, 6.1)	242	1 (−3.2, 5.5)	189	5.1 (0, 10.5)	0.224
ΣDEHP	436	3.1 (−1.3, 7.7)	243	−0.5 (−6.3, 5.6)	193	**7.1 (0.5, 14.1)**	**0.075**
HDL-C							
MEP	436	0.8 (−3, 4.7)	243	2.7 (−2.2, 7.7)	193	−2.8 (−8.6, 3.5)	0.170
MnBP	408	1.2 (−2.8, 5.4)	225	2.4 (−3, 8.1)	183	−0.5 (−6.1, 5.5)	0.997
MiBP	423	0.8 (−4.1, 6)	235	1.3 (−5, 8.1)	188	1.2 (−6.6, 9.6)	0.869
MBzP	431	−0.2 (−4.2, 3.9)	242	−1.7 (−6.6, 3.5)	189	0.3 (−5.9, 6.8)	0.443
ΣDEHP	436	4.3 (−1.2, 10.2)	243	5.5 (−1.8, 13.3)	193	3.1 (−5.1, 11.9)	0.913

*ΣDEHP: molar sum of MEHP, MEHHP, MEOHP. Total and HDL Cholesterol were log transformed to normalize their distributions. We calculated percent change by exponentiating beta coefficients, subtracting by 1 and multiplying by 100. All models are adjusted for child sex, exact age at examination, and maternal characteristics (age at delivery, parity, education, pre-pregnancy BMI, and smoking in pregnancy). Statistically significant associations (P < 0.05) are displayed in bold type*.

Sex modified the associations between child MBzP and leptin (*p*-sex interaction = 0.059), and child MnBP, MBzP, and ΣDEHP and adiponectin (*p*-sex interaction = 0.047, 0.082, and 0.016, respectively) with negative associations in boys vs. positive in girls at 4 years, but none of the associations reached the level of statistical significance (Table 5). In contrast, among boys we observed higher CRP with increases in ΣDEHP whereas in girls the association was positive (*p*-sex interaction = 0.021).

**Table 5:** Sex-stratified associations between maternal and child urinary individual and summed ΣDEHP phthalate metabolites (log_10_ Transformed, in μg/g Creatinine) with leptin, adiponectin and CRP levels in children aged 4 years.

		**All**		**Boys**		**Girls**	
**Maternal metabolites**	***n***	**% Change (95% CI)**	***n***	**% Change (95% CI)**	***n***	**% Change (95% CI)**	***p*-sex interaction**
Leptin							
MEP	187	4.9 (−10.9, 23.3)	101	8.9 (−14.5, 38.5)	86	−2.7 (−22.6, 22.2)	0.567
MnBP	177	6.6 (−11.1, 27.8)	98	16.2 (−14.9, 58.8)	79	2.6 (−18.3, 29)	0.505
MiBP	182	12 (−12.8, 43.8)	100	13.7 (−24.5, 71.3)	82	9.9 (−19.9, 50.7)	0.744
MBzP	159	20.4 (−7.3, 56.3)	84	19.2 (−17.2, 71.4)	75	15 (−23.5, 72.8)	0.807
ΣDEHP	173	17.7 (−3.6, 43.8)	95	21.5 (−7.2, 59.1)	78	6.4 (−23.5, 47.9)	0.559
Adiponectin							
MEP	189	−11.8 (−25.3, 4.2)	103	−15.1 (−32.5, 6.9)	86	−3.4 (−25.2, 24.6)	0.643
MnBP	179	−0.7 (−17.9, 20)	100	−17.9 (−39, 10.4)	79	10 (−15.7, 43.5)	**0.094**
MiBP	184	1.2 (−21.7, 30.7)	102	−0.9 (−32.6, 45.7)	82	0.7 (−29.4, 43.6)	0.931
MBzP	161	−11.8 (−32.5, 15.3)	86	−18.9 (−42.2, 13.9)	75	−3.4 (−39.1, 53.4)	0.213
ΣDEHP	175	1 (−17.7, 24)	97	0.1 (−22.3, 28.9)	78	3.6 (−28.8, 50.7)	0.638
CRP							
MEP	168	25.7 (−17.7, 91.8)	90	21.9 (−31.4, 116.7)	78	51.5 (−24.8, 205.5)	0.94
MnBP	158	34.8 (−16.7, 118.3)	87	−49.1 (−76.7, 11.2)	71	144.3 (26, 373.8)	**0.001**
MiBP	163	1 (−46.3, 90)	89	19.7 (−53.7, 209.6)	74	−14.8 (−66.4, 116.4)	0.682
MBzP	142	−9.2 (−53.1, 75.6)	75	−1.9 (−58.6, 132.2)	67	−13.1 (−72.8, 177.6)	0.87
ΣDEHP	154	1.3 (−39.4, 69.4)	84	−22.8 (−58.8, 44.7)	70	116 (−18.9, 475.1)	**0.053**
**Child metabolites**							
Leptin							
MEP	417	2 (−11.1, 17.1)	230	5.7 (−12.4, 27.5)	187	−0.4 (−19.7, 23.6)	0.652
MnBP	389	2.3 (−11.5, 18.2)	212	0.9 (−18.6, 24.9)	177	2.7 (−16.1, 25.5)	0.719
MiBP	404	4.5 (−12.6, 24.9)	222	−0.3 (−22.2, 27.7)	182	11.5 (−15.6, 47.4)	0.46
MBzP	412	0.1 (−13.7, 16.1)	229	−11.6 (−27.7, 8)	183	16.4 (−7.2, 46)	**0.059**
ΣDEHP	417	−2.3 (−19.4, 18.3)	230	−9 (−30.8, 19.6)	187	6.8 (−19.3, 41.4)	0.376
Adiponectin							
MEP	419	1.4 (−10.3, 14.8)	231	−8.4 (−22.5, 8.4)	188	14.5 (−4.9, 37.8)	0.144
MnBP	391	−0.9 (−13.1, 13)	213	−8.7 (−24.5, 10.5)	178	9.8 (−8.3, 31.5)	**0.047**
MiBP	406	0.2 (−14.4, 17.4)	223	0.7 (−18.5, 24.5)	183	0.8 (−21.4, 29.2)	0.821
MBzP	414	4.8 (−8.2, 19.6)	230	−2.7 (−18.8, 16.5)	184	15.9 (−4.6, 40.8)	**0.082**
ΣDEHP	419	−0.8 (−16.4, 17.8)	231	−21.1 (−38.1, 0.7)	188	24.2 (−2.4, 58)	**0.016**
CRP							
MEP	385	−23.5 (−43.4, 3.3)	211	−24.6 (−49.6, 12.8)	174	−13.5 (−46.8, 40.6)	0.531
MnBP	357	−2.8 (−29.4, 33.8)	193	37.8 (−12.6, 117.4)	164	−34.7 (−58.7, 3.3)	**0.021**
MiBP	372	−26.8 (−50.3, 8)	203	−21.6 (−54.3, 34.3)	169	−35.9 (−65, 17.4)	0.695
MBzP	380	21.5 (−12.5, 68.7)	210	16.5 (−25.5, 82.2)	170	25 (−24.5, 106.7)	0.982
ΣDEHP	385	−12.1 (−42.7, 35)	211	−11.3 (−50.7, 59.9)	174	−8.5 (−52.6, 76.6)	0.862

*ΣDEHP: molar sum of MEHP, MEHHP, MEOHP. Total and HDL Leptin, adiponectin and CRP were log transformed to normalize their distributions. We calculated percent change by exponentiating beta coefficients, subtracting by 1 and multiplying by 100. All models are adjusted for child sex, exact age at examination, BMI z-score, and maternal characteristics (age at delivery, parity, education, pre-pregnancy BMI, and smoking in pregnancy). Statistically significant associations (P < 0.05) are displayed in bold type*.

Effect estimates of the crude models for the associations between phthalate metabolite concentrations and all the obesity-related outcomes under study did not differ substantially from the final models adjusted for child and maternal characteristics (data not shown). The exclusion of preterm newborns and infants with low birth weight did not appreciably change our results (data not shown).

## Discussion

In this prospective cohort study we found that childhood exposure to phthalate metabolites was associated with markers of adiposity and metabolic function and many of these associations were dependent on sex. Additionally, we observed consistent negative associations between phthalate metabolite concentrations at age 4 and systolic and diastolic blood pressure at all ages, overall and in girls or boys separately. To our knowledge this study is the first to examine the relationship between exposure to phthalate metabolites measured prenatally and during childhood with repeated measures of adiposity and a full range of cardiometabolic traits in children.

We observed that child phthalate concentrations were negatively associated with BMI z-score, waist circumference, sum of skinfolds and waist-to-height ratio in boys and positively in girls at age 4 to 6. These findings are consistent with the observations by Yang et al. (27) that, in cross-sectional analyses, found a negative association of child's urine phthalate concentrations with waist circumference and sum of skinfold thicknesses in boys. However, our findings are not consistent with a US study that examined only girls and found that exposure to phthalates at ages 6–8 were associated with a predicted decrease in BMI from the ages of 7–13 (31). We did not observe statistically significant associations between prenatal phthalate metabolites and any of the examined adiposity measures in childhood, consistent with observations from US cohort, where no associations with fat mass in children aged 4–9 years were observed (24), a Mexican study that did not observe any relationships between prenatal phthalate exposure and child BMI after excluding children who had initiated puberty (27) and the HOME study from Ohio that reported no association between prenatal phthalate exposure and child adiposity at age 8. However, depending on age, sex, timing of exposure, and phthalate metabolites, other studies report discrepant findings (25, 26, 28–32). Prenatal exposure to MEP was associated with increased obesity outcomes at ages 5–12 years (30) and increased weight growth velocity at ages 2 and 4 years and increased BMI at 5 years in boys from the French EDEN cohort (26). Furthermore, *in utero* exposure to phthalate metabolites was associated with increased BMI and risk for overweight/obesity between 5 and 12 years of age in the US CHAMACOS study (30). In contrast, prenatal exposure to different phthalate metabolites was associated with decreased BMI z-scores only in girls aged 4–7 years (25), or only in boys aged 4 or 7 years old (28), and 5–7 years old (29).

Our results indicate that that exposure to phthalates may influence adiposity differently in boys and girls. Although still not clear, there are several potential biologic mechanisms underlying associations between phthalates and body size that may be linked to some of the previously observed sex-specific effects. The most plausible mechanism for associations, specifically for body weight and adiposity measures, is that phthalates interfere with peroxisome proliferator-activated receptors (PPARs), which are involved in the metabolism of fat, carbohydrates, and protein (18) and are key regulators of adipogenesis and energy storage, in both rodent and human cell lines (39). Phthalates can activate PPAR-γ resulting in changes in adipocyte differentiation and release of leptin and adiponectin from adipocytes (40). We found some evidence suggesting that sex also modifies the association between phthalates and adipokines. Because early childhood adiposity has been positively associated with leptin levels (41) these sexually dimorphic associations might explain the positive associations that we observed with adiposity markers in girls, however, effect heterogeneity by child sex merit further exploration in larger populations. Moreover, differences in PPAR activity by sex could potentially explain sex-specific associations (42–44). Some phthalates also have anti-androgenic and weakly estrogenic properties (17) and they have been associated with altered sexual differentiation in male rodents (45), lower testosterone levels in an vitro study using human testis (46), and decreased steroid hormone levels in adult men (47) which may explain some of the sex differences reported previously. Another potential mechanism linking phthalates to obesity is through thyroid disruption (14) and negative associations of phthalates with thyroid hormones, insulin-like growth factor I (IGF-I), and growth have been reported in children (48).

Investigations of early life phthalate exposure and cardiometabolic outcomes in humans are scant. Our results suggest that prenatal MEP exposure is associated with lower HDL cholesterol, at age 4 and postnatal MiBP exposure is associated with higher total cholesterol at 4–6 years. The only other study that examined associations of *in utero* and concurrent phthalate exposure with lipid profile reported that prenatal phthalate exposure was not associated with lipid profile at 8–14 years but concurrent exposure was associated with lower total and LDL cholesterol in boys (33).

Moreover, we found that exposure to DEHP metabolites during pregnancy was associated with lower systolic and diastolic BP z-scores and we observed consistent negative associations between phthalate metabolite concentrations at age 4 and systolic and diastolic blood pressure at all ages, overall and in girls or boys separately. These findings are consistent with the only other prospective study that examined prenatal phthalate exposure and blood pressure in childhood and found that ΣHMWPm and ΣLMWPm were associated with lower systolic blood pressure z-scores but only in girls at 4–7 years of age in Spanish INMA cohort (28). However, a recent cross-sectional study of children who participated in the National Health and Nutrition Examination Survey (NHANES), reported positive associations between phthalate exposure and systolic blood pressure in children 6–19 years (23).

There are some biologically plausible mechanisms linking phthalates to increased cardiovascular risk, independent of body mass effects. Laboratory studies have found that phthalate metabolites increase cytokine production (49), while biomarkers of phthalate exposure have been associated with increases in serum markers of inflammation and oxidative stress in adults (50). Recent findings suggest that phthalates may produce increases in low-grade albuminuria in children, a marker of vascular dysfunction associated with chronic kidney and CVD risks (51). Thus, further prospective follow-up of the observed associations will be required to explore whether the associations shown between phthalate exposure and blood pressure persist or reverse at later ages.

The current study has several strengths including the population-based prospective design, our ability to examine the potential effects of phthalate exposure in two critical developmental time periods (pregnancy and childhood), and our comprehensive assessment of adiposity and cardiometabolic outcomes. Limitations of this study include the possibility of exposure misclassification due to the use of a single spot-urine during pregnancy and childhood, and the potential for residual confounding, in particular with respect to unmeasured factors such as parental income or social class. Additionally, as this analysis was exploratory, we have chosen not to control for multiple comparisons, thus we cannot rule out the possibility of some false significant findings.

## Conclusions

Our findings suggest that the effect of early life exposure to phthalate metabolite may influence postnatal growth and adiposity in a sex specific manner and depends on the timing of exposure. Additional longitudinal studies with multiple repeated phthalate measurements throughout childhood and adolescence and diverse study populations are necessary to confirm these findings.

## Author contributions

LC, the principal investigator of the study, and MV conceived and designed the study. MV, TR and KM analyzed data and performed statistical analysis. MV interpreted the results and wrote the manuscript. AM participated in writing the manuscript. All authors made significant contributions to the interpretation of data and participated in drafting and revising the manuscript. All authors have approved the final version.

### Conflict of interest statement

MV has no competing financial interests but she discloses that she receives research funding from Roche, Biogen, and NIH, outside of current study. The remaining authors declare that the research was conducted in the absence of any commercial or financial relationships that could be construed as a potential conflict of interest.
