# Supplementary material for: Association of Early Life Exposure to Phthalates With Obesity and Cardiometabolic Traits in Childhood: Sex Specific Associations
Source: Front Public Health. 2018 Nov 27;6:327. doi: 10.3389/fpubh.2018.00327 (PMC6277685; doi:10.3389/fpubh.2018.00327)
Supplement: Supplementary file 1 [file Data_Sheet_1.docx]

Supplemental Material

**Title:** Association of Early Life Exposure to Phthalates with Obesity and Cardiometabolic Traits in Childhood

**Authors:** Marina Vafeiadi,^1^ Antonis Myridakis,^2^ Theano Roumeliotaki,^1^ Katerina Margetaki^1^, Georgia Chalkiadaki,^1^ Eirini Dermitzaki,^3^ Maria Venihaki,^3^ Katerina Sarri,^1^ Maria Vassilaki,^1,4^ Vasiliki Leventakou^1^, Euripides G. Stephanou,^2^  Manolis Kogevinas,^5,6^ Leda Chatzi^1,7,8^

^1^Department of Social Medicine, Faculty of Medicine, University of Crete, Heraklion, Greece, ^2^Environmental Chemical Processes Laboratory (ECPL), Department of Chemistry, University of Crete, Heraklion, Greece, ^3^Department of Clinical Chemistry, School of Medicine, University of Crete, Heraklion, Crete, Greece, ^4^Department of Health Sciences Research, Mayo Clinic, Rochester, MN, USA, ^5^Barcelona Institute for Global Health (ISGlobal), Barcelona, Spain , ^6^Hospital del Mar Research Institute (IMIM), Barcelona, Spain, ^7^Department of Preventive Medicine, Keck School of Medicine, University of Southern California, Los Angeles, USA, ^8^Department of Genetics & Cell Biology, Faculty of Health, Medicine and Life Sciences, Maastricht University, Maastricht, Netherlands

Figure S 1. GAMS; Sex-stratified adjusted associations (95% CIs) of ΣDEHP phthalate metabolites (log_10_ Transformed, in μg /g Creatinine) at 4 years with systolic and diastolic BP z-scores at 4 years of age. Adjusted child sex, exact age at examination, and maternal characteristics (age at delivery, parity, education, pre-pregnancy BMI, and smoking in pregnancy). ++, observations.


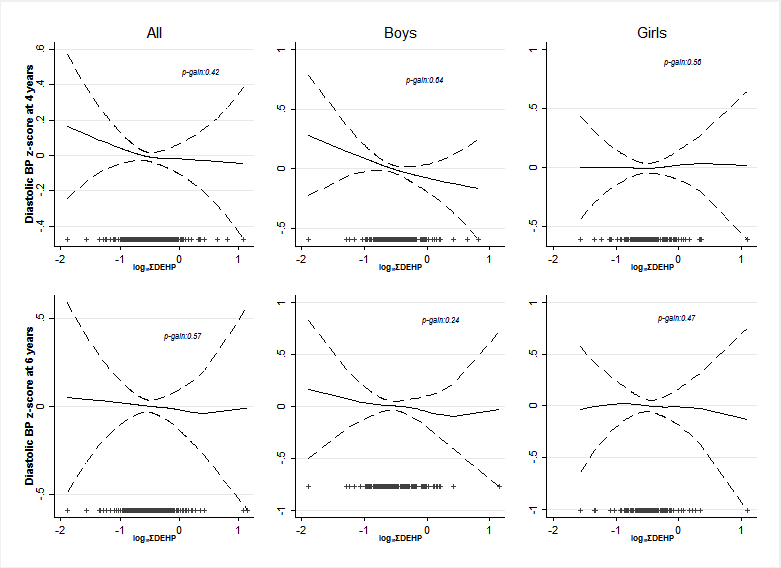


Table S 1. Sex-stratified associations between prenatal urinary individual and summed ΣDEHP phthalate metabolites (log10 Transformed, in μg /g Creatinine) with BMI z-score and adiposity indicators in children aged 4-6 years.

|  |  | All |  | Boys |  | Girls |  |
| --- | --- | --- | --- | --- | --- | --- | --- |
|  | n | β (95% CI) | n | β (95% CI) | n | β (95% CI) | p-sex interaction |
| BMI z-score |  |  |  |  |  |  |  |
| MEP | 220 | -0.09 (-0.28, 0.11) | 123 | -0.1 (-0.32, 0.12) | 97 | -0.09 (-0.45, 0.26) | 0.755 |
| MnBP | 209 | -0.18 (-0.41, 0.05) | 120 | -0.05 (-0.35, 0.24) | 89 | -0.27 (-0.65, 0.12) | 0.312 |
| MiBP | 214 | -0.3 (-0.6, 0.01) | 122 | -0.31 (-0.69, 0.06) | 92 | -0.25 (-0.77, 0.27) | 0.937 |
| MBzP | 186 | -0.13 (-0.44, 0.19) | 104 | -0.24 (-0.58, 0.1) | 82 | 0.06 (-0.54, 0.66) | 0.425 |
| ΣDEHP | 203 | -0.21 (-0.45, 0.03) | 117 | -0.21 (-0.46, 0.03) | 86 | -0.15 (-0.68, 0.38) | 0.893 |
| Waist circumference |  |  |  |  |  |  |  |
| MEP | 219 | -0.29 (-1.47, 0.88) | 122 | -0.15 (-1.54, 1.23) | 97 | -0.51 (-2.57, 1.55) | 0.816 |
| MnBP | 208 | -0.61 (-2.04, 0.82) | 119 | 0.58 (-1.3, 2.46) | 89 | -1.68 (-3.93, 0.57) | 0.123 |
| MiBP | 213 | -0.65 (-2.53, 1.23) | 121 | -1.03 (-3.42, 1.36) | 92 | -0.37 (-3.44, 2.69) | 0.72 |
| MBzP | 185 | -0.79 (-2.73, 1.16) | 103 | -1.21 (-3.44, 1.02) | 82 | -0.14 (-3.64, 3.36) | 0.57 |
| ΣDEHP | 202 | -0.61 (-2.05, 0.83) | 116 | -0.68 (-2.25, 0.88) | 86 | -0.15 (-3.1, 2.81) | 0.742 |
| Sum of skinfolds |  |  |  |  |  |  |  |
| MEP | 212 | 0.19 (-2.82, 3.21) | 118 | -0.04 (-3.67, 3.59) | 94 | 0.19 (-5.07, 5.45) | 0.825 |
| MnBP | 202 | -0.58 (-4.28, 3.13) | 115 | 4.54 (-0.22, 9.31) | 87 | -4.45 (-10.12, 1.22) | 0.015 |
| MiBP | 207 | -1.56 (-6.42, 3.31) | 117 | 0.81 (-5.44, 7.07) | 90 | -4.55 (-12.28, 3.18) | 0.328 |
| MBzP | 179 | 0.22 (-4.73, 5.18) | 99 | 0.31 (-5.66, 6.27) | 80 | -0.58 (-8.83, 7.66) | 0.908 |
| ΣDEHP | 196 | -0.53 (-4.21, 3.14) | 112 | 0.15 (-3.88, 4.19) | 84 | -1.83 (-9.26, 5.59) | 0.618 |
| Waist-to-height ratio |  |  |  |  |  |  |  |
| MEP | 219 | -0.01 (-0.05, 0.03) | 122 | -0.02 (-0.08, 0.05) | 97 | 0 (-0.02, 0.01) | 0.754 |
| MnBP | 208 | **0.1 (0.05, 0.14)** | **119** | **0.19 (0.11, 0.27)** | **89** | -0.01 (-0.03, 0.01) | **<0.001** |
| MiBP | 213 | 0.03 (-0.03, 0.09) | 121 | 0.08 (-0.03, 0.19) | 92 | -0.01 (-0.04, 0.01) | 0.138 |
| MBzP | 185 | 0.04 (-0.03, 0.11) | 103 | 0.06 (-0.05, 0.18) | 82 | 0.01 (-0.02, 0.03) | 0.7 |
| ΣDEHP | 202 | 0.01 (-0.04, 0.06) | 116 | 0.02 (-0.06, 0.09) | 86 | 0 (-0.03, 0.02) | 0.916 |

ΣDEHP: molar sum of MEHP, MEHHP, MEOHP. All models are adjusted for child sex, exact age at examination, and maternal characteristics (age at delivery, parity, education, pre-pregnancy BMI, and smoking in pregnancy). Statistically significant associations (P <0.05) are displayed in bold type.

Table S 2. Sex-stratified associations between child urinary individual and summed ΣDEHP phthalate metabolites (log10 Transformed, in μg /g Creatinine) with BMI z-score and adiposity indicators in children aged 4-6 years.

|  |  | All |  | Boys |  | Girls |  |
| --- | --- | --- | --- | --- | --- | --- | --- |
|  | n | β (95% CI) | n | β (95% CI) | n | β (95% CI) | p-sex interaction |
| BMI z-score |  |  |  |  |  |  |  |
| MEP | 447 | -0.08 (-0.25, 0.1) | 250 | **-0.22 (-0.44, 0)** | 197 | 0.17 (-0.12, 0.45) | **0.051** |
| MnBP | 419 | 0.15 (-0.03, 0.34) | 232 | -0.1 (-0.35, 0.15) | 187 | **0.39 (0.11, 0.66)** | **0.010** |
| MiBP | 434 | 0.12 (-0.11, 0.36) | 242 | **-0.31 (-0.6, -0.02)** | 192 | **0.74 (0.37, 1.1)** | **0.000** |
| MBzP | 442 | 0.17 (-0.01, 0.35) | 249 | -0.02 (-0.26, 0.21) | 193 | **0.42 (0.14, 0.7)** | **0.015** |
| ΣDEHP | 447 | -0.02 (-0.27, 0.22) | 250 | -0.29 (-0.61, 0.03) | 197 | 0.33 (-0.04, 0.71) | **0.016** |
| Waist circumference |  |  |  |  |  |  |  |
| MEP | 446 | -0.66 (-1.78, 0.45)* | 249 | **-1.66 (-3.11, -0.21)** | 197 | 0.91 (-0.83, 2.65) | **0.040** |
| MnBP | 418 | 0.41 (-0.74, 1.56) | 231 | -1.08 (-2.65, 0.49) | 187 | **1.85 (0.18, 3.52)** | **0.011** |
| MiBP | 433 | 0.12 (-1.37, 1.61) | 241 | **-2.04 (-4, -0.09)** | 192 | **3.17 (0.92, 5.42)** | **0.000** |
| MBzP | 441 | 1.04 (-0.11, 2.19) | 248 | -0.2 (-1.75, 1.34) | 193 | **2.6 (0.91, 4.3)** | **0.013** |
| ΣDEHP | 446 | -0.45 (-2.02, 1.11) | 249 | **-2.6 (-4.72, -0.48)** | 197 | 2.14 (-0.14, 4.43) | **0.003** |
| Sum of skinfolds |  |  |  |  |  |  |  |
| MEP | 432 | -1.33 (-6.47, 3.8) | 241 | -4.68 (-12.69, 3.33) | 191 | **5.75 (1.41, 10.08)** | **0.086** |
| MnBP | 404 | 4.53 (-1.22, 10.29) | 223 | 3.36 (-6.29, 13.02) | 181 | **5.04 (0.77, 9.3)** | 0.727 |
| MiBP | 420 | 5.82 (-1.23, 12.86) | 234 | 3.3 (-7.71, 14.31) | 186 | **10.6 (4.96, 16.24)** | 0.227 |
| MBzP | 427 | **7.43 (1.95, 12.9)** | 240 | 6.77 (-1.99, 15.53) | 187 | **8.37 (4.03, 12.72)** | 0.727 |
| ΣDEHP | 432 | 3.8 (-3.91, 11.5) | 241 | 3.18 (-9.36, 15.71) | 191 | **7.55 (1.64, 13.46)** | 0.696 |
| Waist-to-height ratio |  |  |  |  |  |  |  |
| MEP | 446 | 0 (-0.01, 0)* | 249 | **-0.01 (-0.02, 0)** | 197 | 0.01 (0, 0.02) | **0.022** |
| MnBP | 418 | 0.01 (0, 0.01) | 231 | -0.01 (-0.02, 0) | 187 | **0.02 (0.01, 0.03)** | **0.001** |
| MiBP | 433 | 0.01 (0, 0.02) | 241 | -0.01 (-0.02, 0.01) | 192 | **0.04 (0.02, 0.05)** | **0.000** |
| MBzP | 441 | **0.01 (0, 0.02)** | 248 | 0 (-0.01, 0.01) | 193 | **0.03 (0.02, 0.04)** | **0.002** |
| ΣDEHP | 446 | 0.01 (-0.01, 0.02) | 249 | -0.01 (-0.03, 0.01) | 197 | **0.02 (0.01, 0.04)** | **0.006** |

ΣDEHP: molar sum of MEHP, MEHHP, MEOHP. Total and HDL Cholesterol were log transformed to normalize their distributions. We calculated percent change by exponentiating beta coefficients, subtracting by 1 and multiplying by 100. All models are adjusted for child sex, exact age at examination, and maternal characteristics (age at delivery, parity, education, pre-pregnancy BMI, and smoking in pregnancy). Statistically significant associations (P <0.05) are displayed in bold type.*Interaction of exposure variable with child age at examination is statistically significant (P < 0.05).
